# Correlates of adverse childhood experiences among admitted patients with schizophrenia in a referral psychiatric hospital in Botswana

**DOI:** 10.1177/00207640241291500

**Published:** 2024-11-25

**Authors:** Kagiso Bojosi, Anthony A. Olashore, Hlanganiso Roy, Keneilwe Molebatsi

**Affiliations:** Department of Psychiatry, University of Botswana, Gaborone, Botswana

**Keywords:** Adverse childhood experience, Schizophrenia, childhood trauma

## Abstract

**Introduction:**

Schizophrenia is becoming more prevalent globally, particularly in lower and middle-income countries. Adverse childhood experiences (ACEs) are significant risk factors for developing and worsening the disorder. This study aimed to determine the pattern and correlates of ACE among inpatients with Schizophrenia at Sbrana Psychiatric Hospital in Botswana.

**Methods:**

In a cross-sectional cohort study, 128 adult patients diagnosed with Schizophrenia were sampled over a 6-month period. The Adverse Childhood Experiences International Questionnaire (ACE-IQ) and the Positive and Negative Syndrome Scale (PANSS) were used to study ACEs and assess schizophrenia severity. A regression model was used to determine factors that predicted the severity and frequency of admissions, with a significance level set at *p* ⩽ .05.

**Results:**

Participants were mostly males (78.9%), with a mean age of 36.7 (*SD* = 11.01). About 93.8% reported at least 1 ACE, and 56.3% had ⩾4 ACEs. There was a positive correlation between the number of ACEs and positive symptoms (*r*_s_ = .24, *p* < .01) and the general psychopathology score (*r*_s_ = .18, *p* < .05). Having an incarcerated household member (AOR = 2.43; 95% CI [1.02, 5.81]) was associated with PANSS >75. Participants who had experienced physical abuse were more likely to have multiple admissions (AOR = 5.88; 95% 95% CI [1.87, 18.51]).

**Conclusion:**

Our findings highlight the high prevalence of multiple ACEs among patients with Schizophrenia. More interventions are therefore needed to mitigate the risk of ACEs.

## Background

Adverse childhood experiences (ACEs) refer to some of the most intensive and frequently occurring sources of stress that children may suffer early in life (World Health Organization [WHO], 2016). They include abuse, neglect, violence and household dysfunction (WHO, 2016). Childhood experiences have been shown to have a significant contribution to the overall health of an individual well into adulthood (Asmal et al., 2019; Longden et al., 2016; Longden & Read, 2016; Oladeji et al., 2010). ACEs have been linked with the likelihood of lifetime psychiatric disorders such as Schizophrenia (Oladeji et al., 2010).

There is currently overwhelming evidence that highlights the role of psychosocial factors in the onset and progression of schizophrenia beyond just being associated triggers (Read et al., 2008). There is an established link between adverse childhood experiences and Schizophrenia (Oladeji et al., 2010; Popovic et al., 2019; Varese et al., 2012). An increase in frequency or exposure to childhood adversity is associated with an increased risk of developing a psychotic disorder (Oladeji et al., 2010; Varese et al., 2012). Research findings suggest that ACEs affect up to 30% of the general population, but individuals diagnosed with Schizophrenia have a higher likelihood of experiencing ACEs, with reported prevalence rates of up to 94% (Read et al., 2008; Rosenberg et al., 2007; Sideli et al., 2012; Vallejos et al., 2017; Varese et al., 2012). There are conflicting opinions on the relationship between ACEs and Schizophrenia, with some studies suggesting a causal link and others disputing it due to methodological errors (Bendall et al., 2008; Sideli et al., 2012; Varese et al., 2012).

One of the key psychological theories explaining the relationship between ACEs and Schizophrenia is the attachment theory (Chatziioannidis et al., 2019). ACEs result in insecure attachment such as avoidant and disorganised, and these have been found to be more prevalent in patients with psychosis when compared to non-clinical samples (Chatziioannidis et al., 2019; Korver-Nieberg et al., 2015). In addition, they have also been linked with poorer clinical outcomes (Korver-Nieberg et al., 2015).

Exposure to childhood adversity has been associated with multiple brain alterations in studies involving healthy individuals (Cancel et al., 2019). While other environmental risk factors and genetics may be involved, the association between the severity of the adverse experience and grey matter decrease has been found to be stronger in schizophrenia than in unrelated controls (Cancel et al., 2019). For example, a study conducted in Cape Town, South Africa showed that childhood adversity was associated with white matter abnormalities in first-episode schizophrenia (Asmal et al., 2019). Higher levels of sexual abuse and physical neglect during childhood among patients with schizophrenia have been associated with decreased connectivity in emotional processing pathways involving the amygdala (Cancel et al., 2017).

Biological mechanisms mediating the association between ACEs and mental disorders like schizophrenia remains largely unknown. It is widely hypothesised that the main underlying mechanism for brain changes is chronic hypothalamic-pituitary-adrenal (HPA) axis hyperactivation which happens because of childhood adversity (Cancel et al., 2017; Jiang et al., 2019). There is growing evidence that the abnormal HPA axis functioning resulting from ACEs leads to epigenetic changes such as DNA methylation(Jiang et al., 2019; John et al., 2009; Van Os et al., 2020). It is these changes in vulnerable individuals which lead to the expression of the different features of psychotic symptoms in schizophrenia. In addition to the epigenetic changes, ACEs and other environmental factors that affect the immune system, it has been postulated that ACEs may trigger low-grade immune activation, resulting in raised circulating levels of C-reactive Protein (CRP), Tumour Necrosis Factor (TNF) and Interleukin-6 (IL-6; Baumeister et al., 2016). These have the potential to be included as biomarkers in schizophrenia.

Previous studies have shown a high incidence of child and gender-based violence in sub-Saharan Africa, including Botswana (Amone-P’olak & Letswai, 2020; Phillip & Amone-P’Olak, 2019). However, there is a lack of research on Adverse Childhood Experiences (ACEs) in this region, particularly in Botswana, which makes it difficult to understand the correlation between ACEs and Schizophrenia in this population. The goal of the study is to provide data that can be used to develop prevention strategies for schizophrenia and help clinicians identify patterns that may predict severe mental illnesses such as Schizophrenia in Botswana and similar settings. The study is focussed on determining the pattern and correlates of Adverse Childhood Experiences among inpatients with Schizophrenia at Sbrana Psychiatric Hospital in Botswana.

## Materials and methods

### Study design and site

A cross-sectional cohort study was conducted at Sbrana Psychiatric Hospital (SPH) in Lobatse, a town located in the Southern District of Botswana. SPH is the only psychiatric referral hospital in Botswana, making it the most ideal site for the study as it receives referrals from the entire country. The hospital has an average bed occupancy of around 200 and has a 300-bed capacity since it replaced the old Lobatse Mental Hospital in 2009. It offers various services such as in and outpatient psychiatric care, Day Hospital, Psychotherapy, Dietetics, Laboratory, Occupational Therapy and Pharmacy. There are a total of nine inpatient wards, three Female wards and six Male wards.

### Study population and sampling

Our research was conducted on patients who were admitted to Sbrana Psychiatric Hospital with a diagnosis of schizophrenia between October 2022 and March 2023. The inclusion criteria for the study were patients who were 18 years old or older, able to communicate in either English or Setswana and assessed as having the capacity to provide voluntary informed consent using the University of California, San Diego Brief Assessment of Capacity to Consent (UBACC).

Purposive sampling was used. Wards were visited on different days, and all patients in each ward with a diagnosis of Schizophrenia confirmed by the first author were included for screening for eligibility of participating in the study until the desired sample size of 128 was reached. Cochran formula (Cochran 1977) was used for sample size calculation. We adjusted for the population of admitted patients with Schizophrenia based on a previous study at the same facility by Opondo et al. (2018).

### Data collection procedure

Approval to conduct the study was obtained from the Institutional Review Board of the University of Botswana (UBIRB), the Ministry of Health and the management of SPH.

The Principal Investigator visited all the wards on different days. Ward registers were used to identify all admitted patients with a diagnosis of Schizophrenia. These were individually approached and briefed about the study in a private consultation room. Patients who were too acutely ill and could not engage in a meaningful conversation were not involved at the time but revisited later. Those willing to participate in the study were screened against the inclusion and exclusion criteria, starting with confirmation of the diagnosis using the DSM 5 criteria for Schizophrenia by the principal investigator. The purpose of the study was then detailed to those who qualified for inclusion in the study in a language of their choice (English or Setswana). The potential participants were assured of confidentiality. They were then given an opportunity to read a consent document and to ask questions for any clarification. The participants were informed of their right to withdraw from the study at any point and for any reason, and that this would not result in them receiving any adverse treatment. A ward nurse (not involved in the study) was asked to administer the UBACC for capacity to consent. Upon being fully informed about the study and assessed for capacity to consent, willing participants were asked to voluntarily sign two consent forms in a language they preferred between English and Setswana. From there the data collection instruments (ACE IQ and PANNS) were administered.

### Measures

#### Adverse Childhood Experiences International Questionnaire (ACE-IQ)

The ACE-IQ was developed in 2009 in a collaboration between the World Health Organisation (WHO) and the US Centres for Disease Control and Prevention (CDC). It is recognised as an effective tool for studying the potential prevalence of standardised factors of adverse childhood exposure. The questionnaire has been validated in different regions across the world, including Africa and confirmed good validity of the WHO ACE IQ content, reliable internal consistency, satisfactory test-retest reliability, as well as semantic equivalence (Katan, 2019). In a similar low-resource setting in Malawi, the ACE IQ was found to be valid and reliable (Kidman et al., 2019).

The first part of the questionnaire is for demographic information. The questions from the ACE-IQ about childhood experience are grouped into 13 categories: emotional abuse; physical abuse; sexual abuse; violence against household members; living with household members who were substance abusers; living with household members who were mentally ill or suicidal; living with household members who were imprisoned; one or no parents, parental separation or divorce emotional neglect; physical neglect; bullying; community violence; collective violence (WHO, 2011).

Two bilingual experts translated the ACE-IQ to Setswana and back-translated it independently to English, following established protocols (Richter, 2011). The participants chose the language they preferred between English and Setswana.

#### Positive And Negative Syndrome Scale (PANSS) for Schizophrenia

This is widely considered to be one of the best-validated instruments for assessing psychopathology associated with schizophrenia. It is a standardised clinical interview that rates the presence and severity of positive and negative symptoms, as well as general psychopathology, for people with schizophrenia within the past week (Opler et al., 2017; PANSS Institute, 1987). Of the 30 items, 7 are positive symptoms, 7 are negative symptoms and 16 are general psychopathology symptoms. The severity of symptoms for each item is rated according to which anchoring points in the 7-point scale (1 = absent; 7 = extreme) best describe the presentation of the symptom. Although mostly validated elsewhere with good validity and reliability, the PANSS has been widely used in sub-Saharan Africa in low-resource settings like ours (Kumar et al., 2020).

#### Diagnostic and Statistical Manual of Mental Disorders, fifth edition (DSM 5)

The principal investigator who was a final year Psychiatry resident with over 9 years experience working in psychiatry, used the DSM 5 criteria for Schizophrenia, to assess if potential participants met the criteria for schizophrenia. The current mental state and previous history were used to avoid excluding asymptomatic patients. Those patients not satisfying the criteria were excluded from the study.

#### University of California, San Diego Brief Assessment of Capacity to Consent (UBACC)

The UBACC is a 10-item scale that includes questions about understanding and appreciating the information concerning a research protocol. When tested in patients with Schizophrenia, the UBACC was found to have good internal consistency, interrater reliability, concurrent validity, high sensitivity and acceptable specificity (Jeste et al., 2007). Any score less than 14.5 indicates impaired capacity to consent.

### Data analysis

The analysis of the data was performed using IBM SPSS Statistics version 25. Descriptive statistics such as percentages and medians were employed wherever applicable to illustrate the socio-demographic factors. In addition, a chart was used to demonstrate the pattern of ACEs, while chi-square tests were utilised to display its distribution by gender, which was presented with a bar graph. Furthermore, a bivariate model was used to analyse the relationship between the independent variables, including sociodemographic and clinical factors, and the outcome. The measured outcomes are the severity of schizophrenia (PANSS ⩾ 75) and the number of admissions, where 5 or more admissions were defined as multiple admissions. Since the data was non-parametric, a Spearman correlation was used to explore the association between variables such as ACE scores, age at first presentation, number of admissions and PANSS scores. All the variables with a *p*-value of ⩽.2 on bivariate analysis were then entered into the multivariate regression model to explore their relationships with the outcome, which was the severity and frequency of admissions. A single-level multivariate logistic regression was performed while controlling for all the covariates. The Hosmer-Lemeshow goodness of fit test for logistic regression was then conducted, with a good fit being accepted at less than .05, and the level of statistical significance for all tests was set at *p* < .05.

## Results

### Demographic characteristics of participants

The mean age of the participants *(SD)* was 36.71 (11.01). Majority were males 78.9% (101), not in a romantic relationship 90.6% (116), unemployed, 72.7% (93) completed Secondary school and above 52.3% (67; Table 1).

**Table 1.** Demographics by gender.

	Total *N* (%)	Male *N* (%)	Female *N* (%)	*p*-Value
	128 (100)	101 (78.9)	27 (21.1)	
Education				.954
Secondary and above	67 (52.3)	53 (52.5)	14 (51.9)	
Below secondary	61 (47,7)	48 (47.5)	13 (48,1)	
Employment				.203
Employed	35 (27.3)	25 (24.8)	10 (37.0)	
Unemployed	93 (72.7)	76 (75.2)	17 (63.0)	
Relationship				.716
Married or living as a couple	12 (9.4)	9 (8.9)	3 (11.1)	
Single	116 (90.6)	92 (91.1)	24 (88.9)	
Age, years; mean (*SD*)	36.71 (11.01)	35.12 (9.12)	42.67 (15.03)	**.01**

### Clinical characteristics of participants

The median age of first presentation with mental illness was 24 years (16–48). Males presented earlier with a median age of 22 years (16–45) than females, 27 years (16–48), *p* = .032. The lifetime median number of admissions was 4 (1–23) with no statistical significance between males and females. The median ACE score was 4 (0–12). The medians for the PANSS Positive, Negative, General psychopathology and total scores were 22, 22, 32 and 68, respectively. Males, median 13 (7–33), had significantly more negative symptoms compared to females, median 9 (7–21; Table 2).

**Table 2.** Clinical characteristics by gender.

	Total	Male	Female	*p*-Value
Age at index presentation; median (range)	24 (16–48)	22.50 (16–45)	27 (16–48)	**.027**
Number of admissions; median (range)	4 (1–23)	4 (1–23)	3 (1–21)	.990
Total ace score; median (range)	4 (0–12)	4 (0–12)	4 (0–10)	.567
PANSS positive score; median (range)	22 (7–40)	22 (7–40)	23 (7–39)	.294
PANSS negative score; median (range)	22 (7–40)	13 (7–33)	9 (7–21)	**.004**
PANSS; General median (range)	32 (16–70)	31 (16–70)	33 (16–64)	.423
PANSS total; median (range)	68 (30–136)	68 (30–136)	65 (30–109)	.553

Bold values indicates statistical significance at the *p* ≤ .05 level.

### Pattern of ACEs

**Figure 1.**
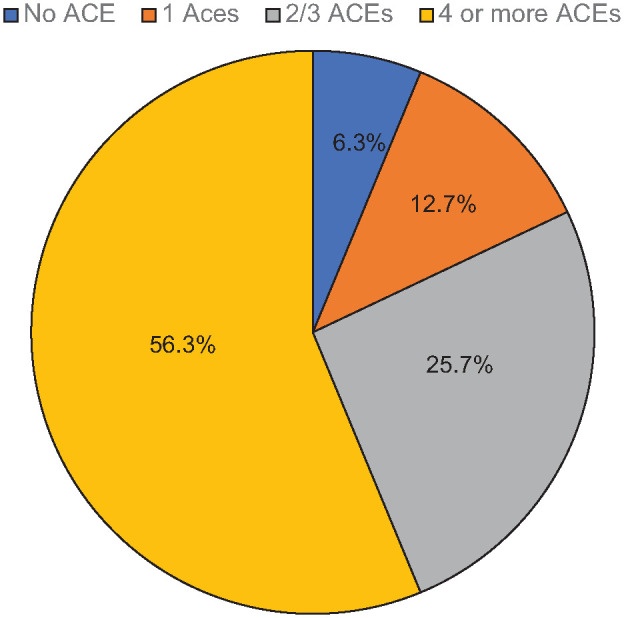
Pattern of ACEs by severity.

Most (93.7%) of the participants reported having cumulatively 1 or more ACE. A majority (56.3%) had 4 or more ACEs. About 25.7% had 2 or 3 while 12.7% reported only 1 ACE (Figure 1).

The most common ACE was Parental separation or Death (69%), followed respectively by household substance abuse (60%) and household member treated violently (58%). The least reported ACE was sexual abuse. More females (37%) than males (8.9%), *p* = .001 reported sexual abuse . None of the participants experienced collective violence. There were no statistically significant associations between the number of ACEs and demographic characteristics.

### Association between Schizophrenia severity, multiple admissions and independent variables

There were statistically significant positive correlations between the age and the number of admissions (*r*_s_ = .21, *p* ⩽ .05) and the age at first presentation (*r*_s_ = .51, *p* ⩽ .01). Age, however, significantly negatively correlated with the total ACE score (*r*_s_ = -.19, *p* ⩽ .05). The number of admissions positively correlated with positive symptoms(*r*_s_ = .20, *p* ⩽ .05; Table 3).

**Table 3.** Spearman correlations between ACE scores and other continuous variables.

	1	2	3	4	5	6	7	8
1. Age at last Birthday	1	0.21*	0.51**	−0.19*	−0.10	−0.03	−0.07	−0.08
2. Number of Admissions		1	−0.35**	0.06	0.20*	−0.13	0.18	0.13
3. Age at last Birthday			1	−0.14	−0.17	0.01	−0.16	−0.16
4. ACE total score				1	0.24**	−0.02	0.18*	0.16
5. PANSS Positive					1	0.16	0.82**	0.85**
6. PANSS Negative						1	0.43**	0.55**
7. PANNS General							1	0.97**
8. Total PANSS								1

* *p* < .05. **p < .01.

Bold values indicates statistical significance at the *p* ≤ .05 level.

There was a statistically significant positive correlation between the number of ACEs and positive symptoms (*r*_s_ = .24, *p* ⩽ .01) as well as general psychopathology symptoms of Schizophrenia (*r*_s_ = .18, *p* ⩽ .05; Table 4).

**Table 4.** Logistic regression model showing the predictors of severe illness (*N* = 128).

Characteristics	*n*	COR	95% CI.	AOR	95% CI.	*p*
Unemployment	93	1.74	[0.73, 4.15]	1.803	[0.73, 4.43]	.199
Present						
Household member incarcerated	28	2.33	[1.00, 5.49]	2.43	[1.02, 5.81]	**.046**
Present						
Parental separation or death	37	0.60	[0.28, 1.30]	0.52	[0.23, 1.17]	.113
Present						

Bold values indicates statistical significance at the *p* ≤ .05 level.

Except for having a household member incarcerated, (AOR = 2.43; 95% CI [1.02, 5.81]), there were no other statistically significant associations between the independent variables (Demographic characteristics, ACE types and ACE severity) and a total PANSS score >75.

Physical abuse predicted multiple admissions (AOR = 5.88; 95% 95% CI [1.87, 18.51]). Participants who did not experience parental separation or death were more likely to have multiple admissions (AOR = 3.43; 95% 95% CI [1.37, 8.60]; Table 5).

**Table 5.** Logistic regression model showing the predictors of multiple admissions (*N* = 114).

Characteristics	*n*	COR	95% CI.	AOR	95% CI.	*p*
Emotional neglect	47	1.87	[0.88, 4.00]	1.79	[0.71, 4.51]	.220
Present						
Household member with mental illness	32	1.87	[0.82, 4.26]	2.44	[0.94, 6.32]	.067
Present						
Parental separation or death	37	2.43	[1.09, 5.42]	3.43	[1.37, 8.60]	**.009**
Absent						
Emotional abuse	36	1.60	[0.72, 3.55]	2.33	[0.69, 7.90]	.176
Present						
Physical abuse	39	4.03	[1.78, 9.15]	5.88	[1.87, 18.51]	**.002**
Present						

Bold values indicates statistical significance at the *p* ≤ .05 level.

## Discussion

The aim of this study was to determine the pattern and correlates of Adverse Childhood Experiences in patients with Schizophrenia at Sbrana Psychiatric Hospital in Botswana. Most of the participants (56.3%) had 4 or more ACEs, while 93.7% reported having at least 1 ACE. The most common ACE was parental separation or death (69%), followed closely by household substance abuse (60%) and household members treated violently (58%). Physical abuse and the absence of parental separation or death predicted multiple admissions. There was some positive correlation between ACE severity and some symptoms of Schizophrenia. Having a household member incarcerated was the only significant association between the independent variables and severe schizophrenia.

The prevalence of at least 1 ACE in this sample is quite high at 93.7%. Although previous studies reported prevalences of up to 94.4% (Mall et al., 2020; Yousef et al., 2022), the prevalence is still significant, especially considering that some of the studies used the binary version of the ACE-IQ, which is less stringent (WHO, 2011). The wide range in the prevalence of ACEs in other studies can also be explained by using disparate tools like the Childhood Trauma Questionnaire (CTQ).

When comparing our results to studies done in our region and other low-resource settings, a case-control study among South African Xhosa people using the CTQ found the prevalence of any childhood trauma among patients with schizophrenia in South Africa to be 94.4% (Mall et al., 2020).

Several studies have found that the presence of one ACE predicts another ACE (Mall et al., 2020; Trotta et al., 2016). Our findings agree with this finding as only 12.7% of the participants experienced one ACE, while a majority had four or more ACEs. Social circumstances that lead to the presence of one are often associated with other ACEs (Gervin et al., 2022; Trotta et al., 2016). It is the cumulative effect of ACEs (usually >4 ACES) that has been associated with poor mental health outcomes, including schizophrenia (Burns, Jhazbhay, Esterhuizen, et al., 2011B; Gervin et al., 2022; Prokopez et al., 2020).

The most prevalent ACE was parental separation or death. In a cross-sectional survey of university students in Botswana, studying the relationship between adverse childhood experiences and depression, (Amone-P’olak & Letswai, 2020) also found parental separation to be the most prevalent ACE. Other studies have found different ACEs to be more prevalent in their settings. In a similar study in Egypt, most of the participants reported having witnessed violence in the household (Yousef et al., 2022). The difference in the pattern may be explained by different socio-cultural and environmental factors which have been noted as significant determinants for the type of ACEs in different areas of the world (Yousef et al., 2022). A possible reason for the high rates of parental loss and separation in our sample could be that most of the participants lost their parents to HIV, which was highly prevalent in the 1990s when most of the participants were younger.

Younger patients were more likely to have more ACEs. This finding is consistent with other studies (Giano et al., 2020; Trotta et al., 2016; Yousef et al., 2022). A possible explanation for this is that the memories of childhood experiences were still relatively fresh compared to the older patients. Younger patients may also be more aware of the ACEs because of access to more education and sensitisation (Giano et al., 2020; Prokopez et al., 2020). ACEs have also been associated with shorter lifespans (Anda et al., 2009; Felitti et al., 1998; Giano et al., 2020). It is therefore possible that patients who had more ACEs did not live long enough to have been represented in this study.

Other studies have found that participants with more ACEs had a younger age of presentation with psychosis (Burns, Jhazbhay, Esterhuizen, et al., 2011; Kasznia et al., 2021; Yousef et al., 2022). Conversely, the correlation between the factors examined in our sample was found to be insignificant. This outcome could be attributed to several factors, including the unreliability of the information pertaining to the initial presentation of patients seeking mental health services. The healthcare system in Botswana relies heavily on primary care clinics and nurses to serve as the first point of contact for most patients. However, the integration with other levels of care is limited, which makes it difficult to access previous records. Although out-patient records are maintained by patients, they are prone to loss. Another plausible explanation could be the prolonged duration of untreated psychosis in patients in low-resource settings like ours since they tend to present late (Farooq et al., 2009; Myaba et al., 2021). Spiritual attribution of cause of mental illness has also been associated with long duration of untreated psychosis as patients and their next of kin often seek help from spiritual and traditional healers before going to psychiatric services (Becker et al., 2019; Burns, Jhazbhay, & Emsley, 2011).

The study reveals a noteworthy correlation between Adverse Childhood Experiences (ACE) scores and positive symptoms and general psychopathology scores. Nevertheless, no such correlation was found with negative and total Positive and Negative Syndrome Scale (PANSS) scores. These findings are consistent with previous studies and confirm a dose-response relationship (Hirt et al., 2019; Prokopez et al., 2020). ACEs are more associated with dissociative symptoms, which in turn are linked with positive symptoms like hallucinations and delusions (Croft et al., 2019). The correlation of positive symptoms and multiple admissions is expected as patients with schizophrenia are mostly admitted due to positive symptoms such as grossly disorganised behaviour and hallucinatory behaviour.

Despite the above correlations, only having a household member incarcerated had a significant association with severe illness (total PANSS > 75). A meta-analysis did not find any specific type of ACE to be a stronger predictor of psychosis than any other (Varese et al., 2012). Other adversity-related variables, such as the frequency and age of exposure, could be more strongly related to psychosis risk than the type of ACE (Varese et al., 2012). Previous studies investigating the relationship between ACEs and Schizophrenia do not use the outcome measure of a defined threshold severity but rather use correlations. The decision to use the cut-off point of 75 to describe severe illness was borne from a study that sought to increase the understanding of what the PANSS score means for both researchers and clinicians (Leucht et al., 2005). In view of the broad dimension of symptoms, simply determining a positive correlation might not be enough, hence the use of an overall severity threshold for practicality and possible real-world application.

The correlation between the severity of psychosis and having a household member incarcerated warrants attention. Although this correlation is tenuous, it is plausible that the experience of having a family member incarcerated could constitute a significant trauma for an already vulnerable individual and influence the progression of the illness. While the present study does not establish a causal relationship, addressing social issues involving family members is advisable, as these issues could indirectly impact the psychosocial well-being of potentially vulnerable family members. Albeit further research is necessary to explore this relationship in more depth, this underscores the importance of considering the social and familial context in the assessment and treatment of vulnerable individuals in the family.

Male participants had statistically significant high scores on the negative symptom subscale. This is consistent with the literature, as males are known to be at a higher risk for negative symptoms compared to females (Li et al., 2022).

For our study, multiple admissions were defined as five or more lifetime admissions in a psychiatric facility. This is similar to some studies investigating ACEs in patients with psychosis (Rosenberg et al., 2007). Although not directly associated with severe illness, physical abuse was the only ACE that was associated with multiple admissions. Psychotic disorders like Schizophrenia are generally characterised by high rates of relapse requiring hospitalisation (Almuqrin et al., 2023). Multiple interacting factors, including ACEs, have been established as contributing to this (Almuqrin et al., 2023). It is plausible that physical abuse could be a factor that persists into adulthood and consequently contributes more significantly to readmissions, as per our findings.

A significant and interesting finding was that participants who did not experience parental separation or death were more likely to be admitted multiple times. A possible explanation for this could be that those patients could have had better care with their parents still around to assist in getting them to mental health services during acute episodes. It is conceivable that parents’ highly expressed emotions may contribute to relapses and inpatient treatment for patients. The emotional state of the parents can significantly affect the well-being of the patients and may lead to a relapse and the need for inpatient care, as patients with overly critical parents have been found to have higher admission rates (Amaresha & Venkatasubramanian, 2012). For example, an old study investigating the experiences of discharged chronic schizophrenia patients surprisingly found that patients who stayed with their parents or wives were more likely to relapse and need readmission than those who lived with their siblings (Amaresha & Venkatasubramanian, 2012; Brown, 1959). It is, therefore, essential to address the emotional well-being of the families of patients, in addition to the patients themselves. Proper attention to the emotional state of the parents can help improve the chances of successful recovery and reduce the likelihood of future relapses.

### Study limitations and strengths

Our study has some limitations that must be discussed. The present study is retrospective in nature and, as such, may be susceptible to recall bias, whereby patients may either under or over-report their adverse childhood experiences. This bias could potentially affect the accuracy and reliability of the findings, highlighting the need for caution in the interpretation of the results. Nevertheless, it has been suggested that retrospective self-reports of childhood adversity by patients with psychosis can be reasonably reliable (Fisher et al., 2011).

As the only psychiatric referral hospital in the country, many patients first go to local district facilities, making it difficult to confirm age at admissions and presentation.

Using purposive sampling ensures that participants meet specific criteria; however, it may introduce selection bias, thereby limiting the generalisability of the findings. Implementing a random sampling approach in future studies could enhance the representativeness of the sample.

Another limitation of the study is the small sample size which was due to a small sample frame. We advise cautious interpretation of our findings as this could have affected the power of the study due to the variability of schizophrenia

This study was limited in measuring the associations between ACEs and Schizophrenia severity due to the varying times of measuring the severity post-admission. Participants assessed earlier may have had more symptoms but fewer ACEs, while those assessed later may have had fewer symptoms but more ACEs. Future studies should assess participants at similar time points to obtain accurate results.

Excluding severely ill patients who couldn’t consent or communicate well might have affected the study’s results. These patients could have experienced more ACEs, which may have influenced correlations. Patients with comorbidities were not excluded due to the small sample frame, this however, could have been a confounding effect which is not accounted for in the study. Also, due to the cross-sectional study design, the direction of causality cannot be determined, although associations can be inferred. One noteworthy aspect of the present study is the employment of the frequency version of the Adverse Childhood Experiences International Questionnaire (ACE-IQ). In contrast to the binary version, the frequency version has demonstrated superior efficacy in evaluating adverse childhood experiences’ outcomes. Although the ACE-IQ was translated to Setswana and back translated to English, the lack of validation of the Setswana translation may still affect its validity and reliability thereby affecting the findings. Another notable strength of this study is that the hospital admits patients from all over Botswana, making the sample population representative of the country’s population.

## Conclusion and recommendations

The impact of Adverse Childhood Experiences (ACEs) on Schizophrenia is a significant psychosocial factor. Our study aimed to assess the prevalence of ACEs among in-patients with Schizophrenia at the sole psychiatric referral hospital in Botswana. The study found a remarkably high prevalence of ACEs in these patients, with most patients having experienced four or more ACEs, which are considered severe. The most commonly reported ACE was parental separation or death. Positive correlations were observed between positive symptoms and general psychopathology scores. Household member incarceration was associated with severe illness, while physical abuse was linked to multiple admissions.

The findings of our research underscore the pressing need for a greater number of psychosocial interventions to counteract the adverse effects of Adverse Childhood Experiences (ACEs). To this end, strategies that encompass all levels of prevention should be instituted, starting with parental education and support for positive parenting and extending to economic assistance for disadvantaged families and communities. Early detection of at-risk children could be facilitated through surveillance and screening programmes, thereby preventing the onset of ACEs.

We recommend future local, prospective studies with larger sample sizes to yield more meaningful results that can inform the development of health policies for interventions in these populations.
